# Testicular ischemia as a result of an incarcerated inguinal hernia containing omentum: a two-case series

**DOI:** 10.1093/jscr/rjac176

**Published:** 2022-04-18

**Authors:** Wendy Chang, Bettina Schulze, Daryl Stephens

**Affiliations:** Department of General Surgery, Mackay Base Hospital, Mackay, QLD, Australia; Department of Urology, Mackay Base Hospital, Mackay, QLD, Australia; Department of General Surgery, Mackay Base Hospital, Mackay, QLD, Australia; Department of Urology, Mackay Base Hospital, Mackay, QLD, Australia

**Keywords:** hernia, general surgery, incarcerated inguinal hernia, acute scrotum, testicular ischemia

## Abstract

Acute scrotal pain is a very common presentation to the emergency room. The most important pathology we must exclude is testicular infarction or testicular ischemia. Here we describe two rare cases of acute scrotum where incarcerated inguinal hernias containing omentum resulted in testicular ischemia/infarction. In Case 1, we describe a rare case in an adult where a large, incarcerated hernia containing omentum along with direct trauma to the testicle resulted in testicular infarction. In Case 2, we describe a 2-year-old boy who presented with left scrotal tenderness due to a left inguinal hernia containing omentum resulting in compromised testicular blood flow. Both patients underwent scrotal exploration. This article also explores the possible pathophysiology of how omentum containing hernias may result in an increased risk of testicular ischemia.

## INTRODUCTION

Acute scrotal pain is a very common emergency department presentation. There is a wide differential that need to be excluded for those presenting with acute scrotal pain including testicular torsion, Richter’s inguinal hernia, Fournier’s gangrene and epididymitis. Inguinal hernia is a very common pathology and repair of inguinal hernias is one of the most common surgical procedures. However, an inguinal hernia containing omentum resulting in testicular infarction is a rare presentation among adults and those >12 months old [1]. The omentum is often regarded as the ‘police-man’ of the abdominal cavity due to its ability to isolate and encapsulate areas of infection and inflammation. One of the functions of the omentum is hemostasis, and it is believed that the omentum assists with prothrombin activation resulting in rapid conversion of fibrinogen to fibrin [1, 2].

## CASES

### Case 1

**Figure 1:**
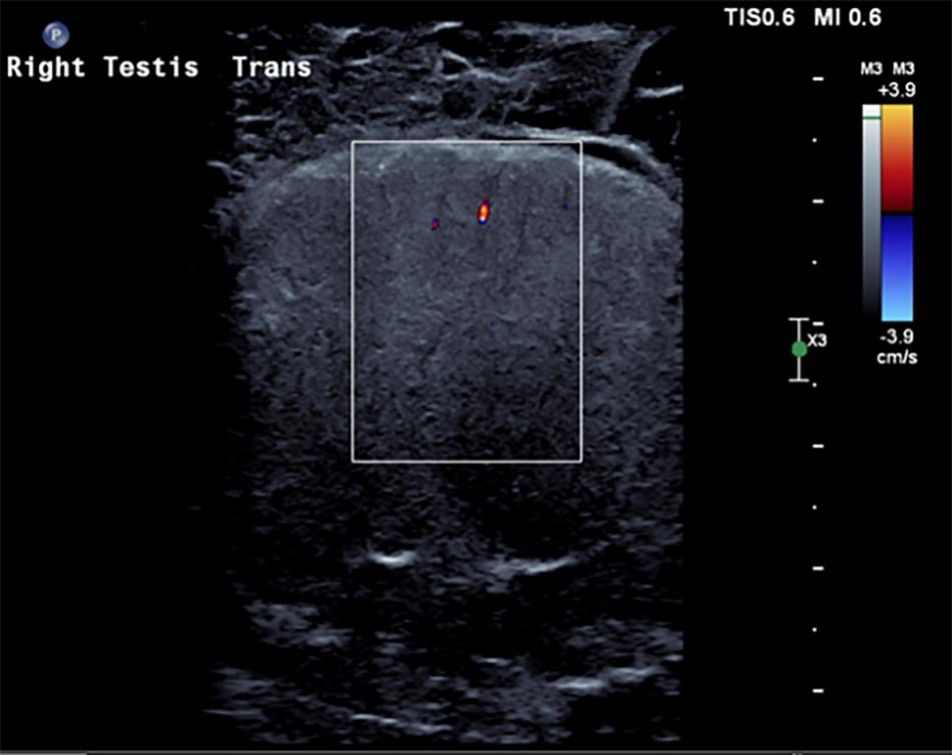
US scrotum showing lack of arterial blood flow to right testicle.

**Figure 2:**
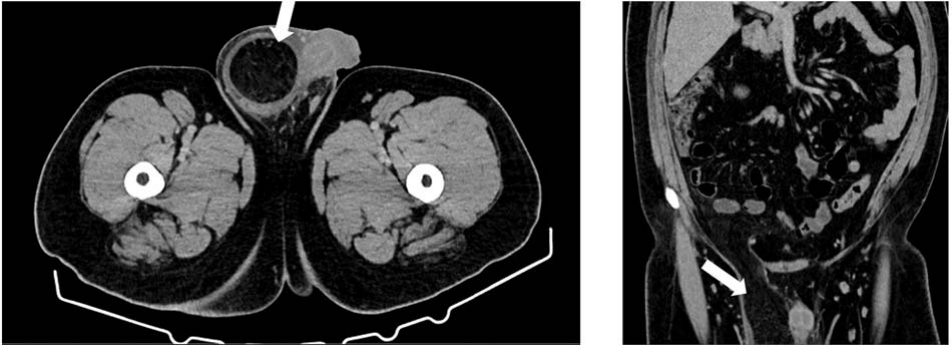
Computed tomography (CT) axial and coronal imaging showing large fat-containing right inguinal hernia (white arrow).

**Figure 3:**
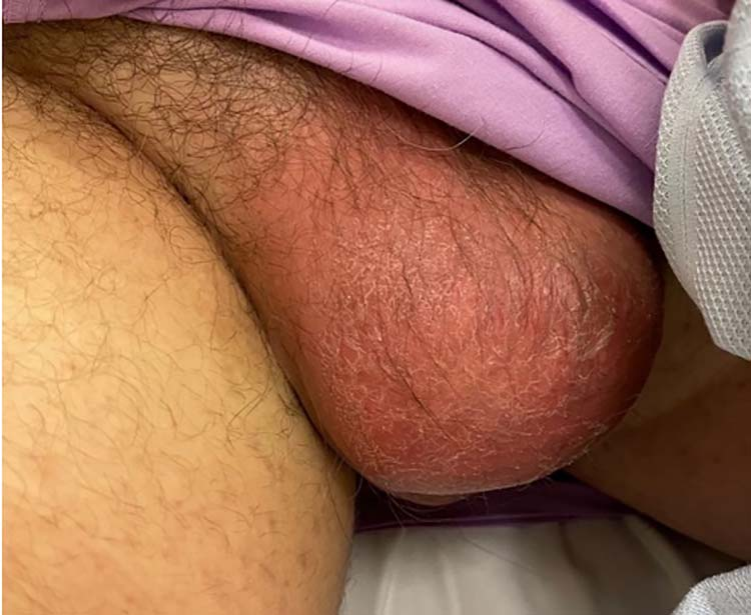
Grossly enlarged and swollen right hemi-scrotum.

**Figure 4:**
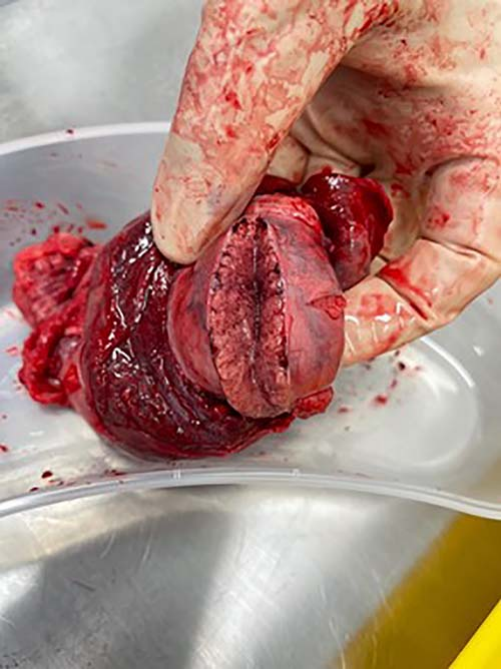
Ischemic, infarcted testicle showing lack of blood flow.

A 27-year-old male presented to the emergency department with acute scrotal pain for 48 hours. He has been having 1-week history of ongoing dull right testicular pain, was diagnosed with epididymal orchitis by his general practitioner and given a week of Augmentin Duo Forte (DF). He denied any history of cryptorchidism. He also has a large right inguinal hernia containing omentum that has been present for 1 year and was awaiting elective repair. Forty-eight hours prior to presentation, his 2-year-old, 20 kg son kicked him in the scrotum. He denied any abdominal pain, dysuria or hematuria. On examination, his right hemiscrotum was grossly swollen, with midline shift of the scrotum, a palpable inguinoscrotal hernia, erythematous, absent cremasteric reflex and a difficult to palpate right testicle. His left testicle had normal examination. He had an urgent scrotal ultrasound (US) showing reduced vascularity of the right testicle, query ischemia (Fig. 1). He then had a computed tomography abdomen and pelvis with contrast showing a large right indirect inguinal hernia containing fat (Fig. 2). On examination, his right hemiscrotum was grossly swollen, with midline shift of the scrotum (Fig. 3), a palpable inguinoscrotal hernia, his scrotum was erythematous, with an absent cremasteric reflex, and a difficult to palpate right testicle. His left testicle had normal examination. He underwent a scrotal exploration with findings of sliding right inguinal hernia with large amount of very stuck and strangulated omentum. The right testicle was found to be infarcted, multiple stabs of the testicle revealed no perfusion, turbid fluid was also noted and the head of the epididymis was necrotic (Fig. 4).

### Case 2

**Figure 5:**
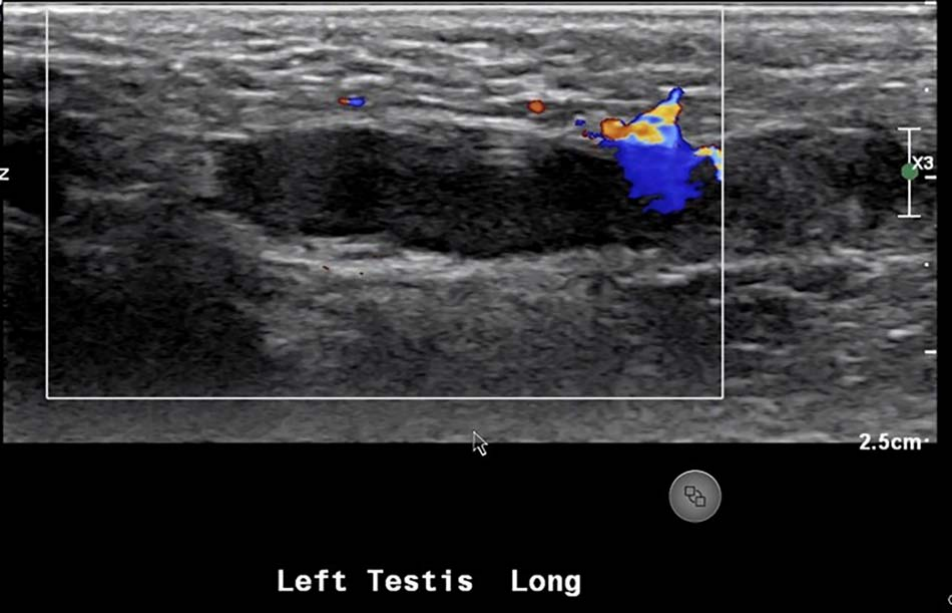
US showing lack of arterial flow into the left testicle.

An 18-month-old boy presented to the emergency room with scrotal pain and scrotal discoloration present for 3 hours prior to ED presentation. His parents denied any malodorous or discoloration of his urine. The child was born at term, and immunizations were up to date. On examination, there was no obvious torsion, and testicles were normal lie. He had a scrotal ultrasound showing reduced blood flow in the left testicle, left epididymitis and torsion could not be completely excluded (Fig. 5). He underwent scrotal exploration with findings of normal left testicular lie with no evidence of torsion of the spermatic cord and the epididymis is within normal limits; however, a small hernia containing healthy omentum was identified. The hernia was reduced and repaired with 3-0 vicryl suture.

## DISCUSSION

Acute scrotal pain is a very common emergency department presentation. Differentials for acute scrotal pain include testicular torsion, epididymo-orchitis, Fournier’s gangrene and scrotal trauma. Inguinal hernia is also a very common pathology and repair of inguinal hernias is one of the most common surgical procedures. However, an indirect inguinal hernia with omentum causing testicular infarction/ischemia is a rare presentation in adults.

The majority of indirect inguinal hernias are congenital and are the result of a patent processus vaginalis into which the abdominal viscera can herniate. In Case 1, our patient had an incarcerated hernia with incarcerated omentum. Our patient presented with 1-week history of dull right testicular pain, which could indicate the beginning of vascular compromise to the right testicle. His son had also kicked him in the scrotum prompting him to present acutely. His ultrasound showed he had a complicated large hydrocele and poor blood flow. Testicular infarction due to an incarcerated inguinal hernia in adults is an extremely rare event; there have only been three cases documented in literature [1–3]. In Case 2, a 2-year-old male who also had a left inguinal hernia containing omentum which resulted in reduced blood flow to his left testicle as confirmed on US scrotum. Incarcerated/strangulated omentum in a hernia causing acute scrotum in non-infant pediatric population is an extremely rare event [1, 3, 4], and as in our case often not noticed until surgical exploration.

In both cases, both patients had inguinal hernias containing omentum resulting in testicular infarction which is rare there is a paucity of cases documented in the adult population and has been documented more commonly in infants [1]. According to Hill *et al*. [1], the proposed mechanism of action resulting in testicular infarction occurs when the incarcerated hernia contents cause enough pressure against the spermatic cord resulting in venous obstruction of the pampiniform plexus. Venous obstruction results in venous thromboses and hemorrhage in the pampiniform plexus [1, 2].

Here we would like to further explore on the pathophysiology in regards to the omentum. What is it about the omentum that causes poor perfusion? The omentum is an immunological organ situated in our peritoneal cavity. It is often referred to as the ‘guardian of the abdomen’. The omentum stores large amount of fat known as omental adipose tissue. Omental adipose tissue plays a crucial role in local immunological response. It contains a large amount of B and T lymphocytes and organizes in clusters called milky spots. Omentum with milky spots actively migrates to areas of inflammation. This results in volume expansion of the omentum in response to foreign particles and inflammation [5]. The expansion and increased edema of the omentum may result in compression of spermatic cord and vascular structures resulting in blood flow impairment to the testicle; however, plasminogen activation inhibitor-1 (PAI-1) activation may also play a role.

Plasminogen activator inhibitor-I (PAI-1) is part of the fibrinolytic system. PAI-1 in particular is a serine-protease inhibitor that inhibits fibrinolysis resulting in increased thrombosis. It is stimulated by interleukin-6, tumor necrosis factor alpha and transforming growth factor beta [5], all of which are elevated during the inflammatory process. Using immunohistochemistry technique, Yildiz *et al*. [6] compared levels of PAI-1 in omental tissue and subcutaneous fat. Tissue samples were obtained from patients who underwent laparotomies. Their results showed significant elevation in expression of PAI-1 in omental adipose tissue when compared with subcutaneous adipose tissue.

In our cases, both our patients had an inguinal hernia that contained omentum. One can draw the conclusion that because of involvement of the omentum it predisposed our patients to develop testicular infarction resulting in one of our patients requiring an orchidectomy. The key lessons for our cases are: male patients with inguinal hernias containing omentum should be categorized as needing an urgent hernia repair in order to maintain testicular viability and those presenting to the emergency department with incarcerated inguinal hernia should receive a scrotal Doppler ultrasound to exclude testicular ischemia.
